# Sex-Specific Differences in Glioblastoma

**DOI:** 10.3390/cells10071783

**Published:** 2021-07-14

**Authors:** Anna Carrano, Juan Jose Juarez, Diego Incontri, Antonio Ibarra, Hugo Guerrero Cazares

**Affiliations:** 1 Department of Neurologic Surgery, Mayo Clinic, Jacksonville, FL 32224, USA; Carrano.Anna@mayo.edu; 2 Centro de Investigación en Ciencias de la Salud (CICSA), FCS, Universidad Anáhuac México Campus Norte, Huixquilucan 52786, Edo. de México, Mexico; juanjojvw96@gmail.com (J.J.J.); incontri.diego897@gmail.com (D.I.); jose.ibarra@anahuac.mx (A.I.)

**Keywords:** glioblastoma, glioma, sex differences, neuro-oncology

## Abstract

Sex differences have been well identified in many brain tumors. Even though glioblastoma (GBM) is the most common primary malignant brain tumor in adults and has the worst outcome, well-established differences between men and women are limited to incidence and outcome. Little is known about sex differences in GBM at the disease phenotype and genetical/molecular level. This review focuses on a deep understanding of the pathophysiology of GBM, including hormones, metabolic pathways, the immune system, and molecular changes, along with differences between men and women and how these dimorphisms affect disease outcome. The information analyzed in this review shows a greater incidence and worse outcome in male patients with GBM compared with female patients. We highlight the protective role of estrogen and the upregulation of androgen receptors and testosterone having detrimental effects on GBM. Moreover, hormones and the immune system work in synergy to directly affect the GBM microenvironment. Genetic and molecular differences have also recently been identified. Specific genes and molecular pathways, either upregulated or downregulated depending on sex, could potentially directly dictate GBM outcome differences. It appears that sexual dimorphism in GBM affects patient outcome and requires an individualized approach to management considering the sex of the patient, especially in relation to differences at the molecular level.

## 1. Introduction

Glioblastoma multiforme (GBM) is the most frequent primary central nervous system (CNS) cancer, accounting for 45.2% of malignant CNS tumors and 55% of all gliomas [1,2]; if untreated the median survival is 3 months [2]. Based on the World Health Organization (WHO) Classification, GBM corresponds to grade IV astrocytoma and remains the most aggressive diffuse glioma of astrocytic lineage despite the best available therapy. Current treatment consists of surgical resection followed by radiotherapy with concurrent temozolomide and chemotherapy cycles [2]. Despite the latest advances in basic research and 12 randomized clinical trials in the past 5 decades, GBM still has a poor prognosis with a low overall survival [1,3]. Even after standard resection, radiotherapy, and chemotherapy, GBM carries a median survival rate of 12 to 15 months, and only 5.5% of all patients survive longer than 5 years [1].

GBM is characterized by hypercellular anaplastic glioma cells with high mitotic activity and the presence of necrosis and microvascular proliferation [4]. A distinction between primary and secondary GBM is important for both clinical and biological research. Primary (de novo) tumors manifest rapidly and without evidence of less-malignant precursor lesions. This subtype accounts for 80% of GBM and mostly occurs in older patients (mean age, 62 years). Secondary tumors develop more slowly, from low-grade (WHO grade II) or anaplastic (WHO grade III) astrocytoma. This subtype occurs more often in younger patients (mean age, 45 years) [2,3].

GBM has an average annual age-adjusted incidence rate of 3.21/100,000 population [5]. In general, GBM evolves through different genetic and molecular pathways, affecting patients at different ages and having distinct outcomes resulting from sex-specific features. During the past few decades, numerous studies have identified several prognostic factors in patients with GBM. Clinical factors such as age at diagnosis, race, Karnofsky Performance Status score, Mini-Mental State Examination score, and extent of surgical resection are all prognostic factors for overall survival in high-grade gliomas [1,6]. Although sex-specific effects in incidence, disease phenotype, and outcome are well described in GBM, few insights are available to distinguish male and female GBM patients at the molecular level or allow specific targeting of these biological differences [7]. This review describes several differences in GBM between men and women with regard to epidemiology, disease phenotype, genetic/molecular factors, and outcomes. Within the disease phenotype, we reviewed sex differences in clinical presentation and the ways in which hormones and different metabolic pathways, as well as the immune system, interact differently in GBM. Regarding genetic and molecular mechanisms, our focus was on how different genes, specific pathways, sequence variations, and epigenetic modifications differ between men and women with GBM. The goal was to understand the specific pathologic mechanisms of GBM for men and women, as well as to determine why this tumor shows such different presentations and outcomes between sexes.

## 2. Epidemiology

Incidence rates for primary malignant tumors of the CNS have consistently increased over the past several decades because of better diagnostic tests, better evaluation of patients, and possibly the concomitant increase in associated risk factors in society [8,9]. In the United States, GBM has an average annual age-adjusted incidence rate of 3.21/100,000 population [5], with a constant growth of 3% per year for both men and women [10]. The Central Brain Tumor Registry of the United States (CBTRUS) Statistical Report from 2011–2015 indicated a total of 57,805 patients with GBM in the United States [5]; the most recent CBTRUS Statistical Report (2013–2017) indicated 60,056 GBM cases in 5 years, with an annual incidence of 12,011 cases [11]. Moreover, GBM has the highest incidence rate among CNS malignant tumors, followed by diffuse astrocytoma and lymphoma [5].

The most obvious difference associated with sex in GBM is its incidence: the incidence of GBM is 1.6 times higher in men than women. Whereas low-grade glioma incidence is nearly similar in men and women, malignant brain tumors, including GBM, occur more often in men. Specifically to GBM subtypes, primary tumors are more frequently seen in men, whereas secondary tumors occur more frequently in women. In human and animal studies, women and female animals have also had longer survival and better outcomes, even after accounting for extent of resection, treatment, and age [1,2,3,6,7,12,13,14,15,16,17,18]. When studying GBM recurrence it is important to note that recurrence is inevitable, occurs in the short term, and shows no clear difference based on sex. The only associated sex difference in recurrence is that left temporal tumors have a shorter recurrence time, and these are more commonly seen in men [19,20]. In contrast, treatment for recurrent GBM does differ by sex [18]. In a multivariate analysis, tumor-specific therapy for first and second recurrences was administered more frequently in men than women, but the effect of this treatment on survival is not yet clear because overall long-term survival is longer in young women [18].

In the past few decades, many studies have focused on the close relationship between age and GBM incidence. We now know that primary GBM predominantly affects older patients, whereas secondary GBM is more frequently seen in younger ones. However, sex-specific effects in GBM incidence rate are also important. Differences in incidence and mortality rates by sex suggest both biological and environmental variables.

Although retrospective studies indicate a higher incidence and worse survival of male GBM patients compared with women, the causes of these differences are poorly understood. Most of the large-scale analyses that exist have combined data from both sexes. These merged data could possibly hide relevant information regarding sex-specific effects on GBM survival [7]. Mechanistic studies are needed regarding the potential causes of sex differences in GBM. These studies should integrate observations at genomic, proteomic, and metabolomic levels.

## 3. Disease Phenotype

Knowing and identifying characteristics that differ between men and women in GBM is extremely important, because it helps physicians and researchers to offer specialized management, investigate new treatments, and understand the disease from a sex-specific perspective. The multiple differences between men and women in terms of clinical features, hormones, metabolism, immune system, genetic and molecular mechanisms, outcomes, and neurogenic niches in relation to GBM pathology are briefly summarized in Table 1.

### 3.1. Clinical Features

GBM is commonly located in the supratentorial region and rarely seen in the cerebellum and spinal cord [2]. Segregation of tumor location data by sex shows differences in the incidence rate between women and men [22]; one population-based study reported a higher GBM incidence in men than women at every anatomic subsite except for the posterior fossa [19]. Respectively, women have a higher incidence in the right temporal lobe, whereas in men the left temporal lobe is more involved. These differences extend to involvement of the right and left periventricular frontal regions for women and men, respectively [21]. In contrast, comparison of temporal vs. frontal lobe involvement in men showed a higher incidence of GBM in the frontal lobe, followed by the left temporal lobe. The higher incidence of frontal lobe GBM in men, localized proximal to the ventricle [22], gives some insight into possible sex-specific effects of GBM near the subventricular zone (SVZ).

**Figure 1:**
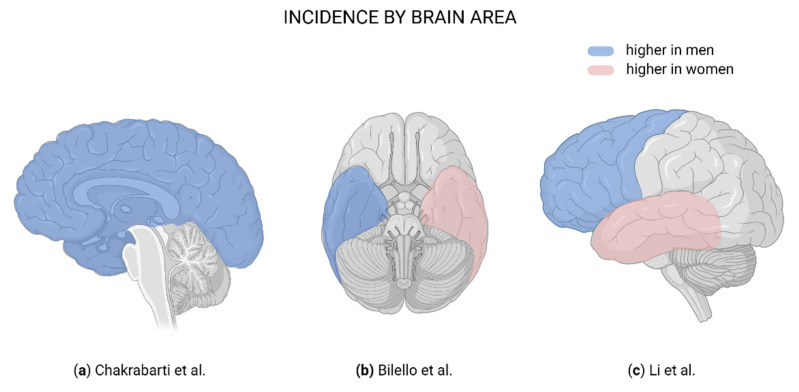
Brain regions affected in glioblastoma. (**a**) Blue indicates a higher male:female ratio in incidence [19]. (**b**) Blue indicates more frequently affected in men; pink, more frequently affected in women [21]. (**c**) Blue indicates more frequently affected in men; pink, more frequently affected in women [22]. Created with BioRender.com.

Even though GBM in the temporal lobe is known to be associated with prolonged overall survival and progression-free survival in the general population, data are still lacking on precise differences in tumor location between sexes and how location affects overall survival in each sex [20,22]. Studies demonstrate that sex may also influence GBM location in the brain (Table 2; Figure 1), which could directly affect sex-specific incidence, recurrence, and survival rates. 

Epileptic seizures are common symptoms at presentation and found to be related to both tumor grade and tumor location, although few studies have investigated the molecular mechanisms underlying epileptogenesis in glioma [44]. A retrospective study reported a higher incidence of seizure in males (58%) than in females (32%) independent of tumor location. Seizures are also slightly more probable for left hemisphere tumors in females, while there is no such distinction in males [45].

Regarding the presence of GBM symptoms, women report a higher intensity and frequency of fatigue, whereas lower fatigue is more common in men, regardless of GBM treatment response [46]. 

Larger tumor size and larger areas of necrosis have been reported in female than male patients with GBM [21]. In addition, female patients with high volumes of necrosis have shorter survival than men with the same conditions [47]. In contrast, increased cell proliferation, vasogenic edema, and necrosis have been demonstrated with magnetic resonance imaging in both men and male rats with GBM [48,49]. 

Although there is enough evidence regarding different GBM incidence rates further studies should address in detail clinical and symptomatic aspects differing between men and women diagnosed with GBM.

### 3.2. Hormones

Sex hormones influence the pathogenesis and outcome of GBM tumors. In women, large-scale studies have documented a strong relationship between estrogens and their neuroprotective effects [47]. In contrast, testosterone has recently gained interest in the field of GBM tumorigenesis because this sex hormone has been suggested to have an important role in the male predominance of the disease [8].

Endogenous estrogens are neuroprotective in various neurologic disorders, including in the development of brain tumors and their growth control [47]. A study using data from the New York State Cancer Registry showed that differences in incidence of GBM by sex (greater in males) begin around the age of menarche in females, are greatest around menopause, and decrease thereafter [8]. Moreover, in a study of the Surveillance, Epidemiology, and End Results Program database, premenopausal women with GBM showed longer survival than men, and this difference disappeared postmenopausally [14]. Postmenopausal women are at higher risk for GBM than premenopausal women [8]. Additionally, late menarche and early menopause (shorter exposure to female hormones) are associated with increased glioma risk [24]. These results suggest a highly protective effect of female hormones in GBM. However, an age-related factor is also involved which gives older women a higher risk of GBM.

Some evidence suggests that sex hormones are able to cross the blood-brain barrier [21] and that estrogens confer a protective effect for both GBM incidence and survival [25]. The effect of menopausal hormone therapy in decreasing the risk of glioma has been previously studied, but the results remain controversial. Two different case-control studies, of 507 and 212 patients with glioma, used unconditional logistic regression analysis and had similar results. The first study showed that older age at menarche was associated with an increased risk of glioma (odds ratio [OR], 1.65; 95% CI, 1.11–2.45), and a stronger association was observed in premenopausal compared with postmenopausal women, concluding that later age at menarche increases the risk of glioma [50]. The second study also showed an increased risk of glioma with older age at menarche (OR, 1.9), but age younger than 20 years at the birth of the first child was associated with a decreased risk of glioma (OR, 0.43). In addition, exogenous hormone use (oral contraceptives and hormone replacement therapy) was associated with a lower risk of glioma [51].

Another study showed that reproductive factors were not associated with gliomas, especially for non-GBM tumors, but inverse associations were observed for the use of both oral contraceptives and postmenopausal hormone therapy, indicating that the protective effects are limited to exogenous hormone therapy only [52]. These findings suggest a possible association of early-life hormonal exposure with risk of glioma, along with the protective effects that exogenous hormone therapy could have on gliomas and that may be applicable to GBM tumors. In contrast, a Canadian study showed a slightly increased risk of glioma in women with a late age at menarche (66%), compared with 64% in those with early age at menarche, but the use of hormone therapy had no association with an increased glioma risk [53]. Similarly, a population-based case-control study in Sweden found no increased risk of gliomas due to exogenous estrogen use [54], and a prospective study in different CNS tumor types showed that the use of hormone replacement therapy, duration of use, and hormonal constituent did not vary by type of CNS tumor, including gliomas [54]. 

Most of these studies support an increased risk of glioma with older age at menarche, but the evidence regarding the use of exogenous hormone therapy is still split, as some results show a decrease in the risk of glioma [50,51,55], while others show no association [52,53,54]. In addition, the Million Women Study concluded that there was no association between current or past use of hormone therapy and glioma risk, but the use of estrogen alone was associated with a minimal but significantly higher risk [54,56]. Studies evaluating the use of oral contraceptives and the risk of gliomas are limited. Some studies found a decreased risk, but no relationship was established with treatment duration [51,57]. It is important to highlight that although the previously mentioned studies have evaluated the risk of gliomas developing, these studies were not specifically focused on GBM.

Even though some authors argue that female hormones are protective against GBM development, there might be other explanations. The proposed benefit derived from exposure to female hormones may actually be the result of protection from the detrimental effects of exposure to male hormones [8]. Castration of male rats led to fewer rats developing glial tumors and longer survival when tumors did develop [58]. Because the upregulation of androgen receptor (AR) expression promotes GBM tumorigenesis, AR has been implicated as a cause of the higher incidence of GBM in adult men [59]. Interestingly, genetic silencing of AR, as well as its pharmacologic inhibition, induce GBM cell death in vivo and in vitro [23,60]. Moreover, an increase in testosterone levels has been reported in GBM patients, which suggests that a testosterone-activated AR signaling pathway has a key role in GBM proliferation, migration, and invasion [23,61].

Greater activation of the mitogen-activated protein kinase (MAPK) pathway has been observed in multiple areas of the male brain. Interestingly, estrogens suppress MAPK activity in a sex-dependent manner. Female astrocytes show a greater sensitivity to the MAPK pathway inhibitory effects of estradiol than male astrocytes [62]. This might be explained by the higher aromatase expression and estradiol formation in female astrocytes than male astrocytes. Aromatase produces estradiol in the brain from testosterone, potentially making women more sensitive than men to the effects of estradiol and testosterone [62]. Estrogen also increases survival in GBM female rats, and this survival advantage is lost after oophorectomy and restored by estrogen replacement therapy [14,63]. Interestingly, other studies using exogenous estradiol delivery in a model of GBM reported an increase in tumor cell apoptotic activity and survival in both male and female rats [14,47].

Recently, some studies using a model of mesenchymal GBM showed that the effect of sex on malignant transformation was evident in the absence of sex hormones, thus providing evidence that GBM sex differences are independent of hormonal activation effects [29]. Although the previously mentioned studies reported estrogen exposure as a protective factor for GBM development in females and detrimental effects of testosterone and AR upregulation on GBM tumorigenesis, other studies indicate no effect of hormonal action in GBM sex differences [7,29]. The male predominance of GBM exists not only in adults but also in pediatric patients [64]. This provides more evidence that sex-specific effects in GBM tumorigenesis are obvious and could possibly be due to a combination of factors including sex hormones and cellular intrinsic differences between the sexes.

The protective effect of estrogen exposure on GBM development and pathogenesis has been well studied, as has the detrimental effects of the upregulation of AR and testosterone on GBM tumorigenesis and its association with a higher incidence in men. These differences are important because they are sex specific and not only explain differences in pathogenesis between sexes but also possibly provide future targets for therapies. However, it is important to mention that sex hormones alone are not the only factors associated with these sex differences in GBM.

### 3.3. Metabolism

Because metabolism is a critical factor required for tumor survival and tumorigenicity, metabolic changes have emerged as a possible mechanism to explain the sex disparity seen in brain cancers, including GBM [28,65].

The most prevalent form of metabolic disease in humans is diabetes. Diabetes is characterized by hyperglycemia, which is also a risk factor for tumor development, because most cancers present with aberrant glucose metabolism (aerobic glycolysis). Despite this association, epidemiologic studies have noted an inverse relationship between type 2 diabetes and GBM, whereas no direct relationship with type 1 diabetes has been reported. Type 2 diabetes in men has been associated with a lower incidence of GBM, but this difference has not been seen in women [66,67]. Even though the mechanism of this relationship is not yet understood, it has been linked to low AR and testosterone levels in men with type 2 diabetes, which highlights the important relationship of metabolic and hormonal changes between sexes and the impact of both on GBM pathogenesis. In addition, even male and female embryos exhibit differences in glucose metabolism and lactate production, being twice as high in male vs. female embryos [64].

These alterations in glucose metabolism are important to mention because they are required for cancer growth. A recent study identified male-specific decreased survival resulting from glycolytic gene overexpression, and women with high glycolytic gene expression survived longer [28]. These data suggest that glycolytic metabolites could possibly stratify survival by sex in GBM. Moreover, enhanced glucose uptake and its conversion to lactate, despite the presence of available oxygen, is observed in cancer cells and is correlated with higher tumor survival. Metabolic reprogramming and utilization of alternative energy sources allow tumor cells to survive in unsupportive microenvironments [68]. Several studies demonstrated that, beside glucose, cancer cells also exploit the mitochondrial tricarboxylic acid cycle (TCA cycle) and oxidative phosphorylation. Mitochondria present a strong sex-dimorphism as they are exclusively maternally inherited. The activity of the mitochondrial enzymes citrate synthase, succinate dehydrogenase, and mitochondrial reductase are significantly higher in females [69]. However, despite their higher respiratory rate, female mitochondria accumulate significantly lower levels of ROS [70] and, consequently, less oxidative damage.

Fatty acids oxidation is also emerging as a key metabolic pathway for metabolic reprogramming in GBM [70,71]. Several studies suggest that males mostly rely on glucose and amino acid utilization, while females appear to favor lipid substrates for energy metabolism [72]. Whether this is the case for GBM cells as well remains to be elucidated.

It is well known that metabolism differs between men and women and that glucose metabolism and metabolic reprogramming most likely are a major contributor to GBM pathogenesis and other brain tumors being more common in men. These differences most likely involve cell-intrinsic growth mechanisms that modify the cellular environment, cell migration, and invasion and could possibly have a relationship with the higher incidence of brain tumors and worse prognosis in men. It is not well determined how different metabolic pathways, especially glucose metabolism, affect survival and prognosis on the basis of sex; therefore, future studies must focus on understanding metabolic sex disparities regarding brain tumor biology.

### 3.4. Immune System

The immune system has an essential role in sex differences in the nervous system and its development [66]. Differences in chromosomal composition and sex hormones between sexes influence the immune response, resulting in more vigorous immune responses in women than men [67]. Emerging literature reveals how sex differences in brain disease prevalence, clinical features, and outcomes might be due to sex differences at the immunologic level [73]. Information is currently limited regarding the role and sex differences of the immune system in GBM incidence but is of recent interest.

Microglia are the principal immune cells of the brain, and microglia/macrophage infiltration is a well-established characteristic of GBM, which suggests a role for innate immunity in the GBM pathologic mechanism. Expression of specific immune-associated molecules is correlated with GBM overall survival. Glioma-associated macrophages and microglia (GAMs) have been implicated in tumor proliferation, migration, invasion, and survival [74,75,76,77]. GAMs also participate in tumor escape from antiangiogenic therapy and represent a potential biomarker of resistance and poor survival among patients with recurrent GBM [78]. The prognostic impact of GAMs in gliomas does not depend on the total amount of GAMs but on their acquired functional phenotype [79]. Specifically, the presence of anti-inflammatory M2 GAMs has been associated with an unfavorable prognosis in high-grade gliomas [32] as well as a poor response to radiotherapy [80]. M2 GAMs release immunosuppressive cytokines such as transforming growth factor β into the GBM microenvironment, which prevent myeloid cells from inducing a coordinated immune response against the tumor [77,81]. Microglia function can be differentially modulated by estrogen in men and women. Although male and female microglia express both estrogen receptor subtypes, α and β [82], estrogen exerts a sexually dimorphic effect on proinflammatory cytokine interleukin (IL) 1β production, promoting a proinflammatory response in female microglia and inducing an anti-inflammatory effect on microglia in men. One study reported conflicting results regarding differences in microglial gene expression between men and women, in which men showed higher expression of inducible nitric oxide synthase in the tumor, indicating an M1 phenotype [83]. These findings indicate a complex interaction between GBM cells and GAMs in different sexes.

Similar dimorphic behavior in the production of cytokines is observed for astrocytes, another important cellular effector of the CNS immune response. For instance, compared with those in women, astrocytes in men produce more IL-1β, IL-6, and tumor necrosis factor α proinflammatory cytokines associated with glioma growth [29]. This divergence could contribute to the differences in GBM growth rates between sexes. Additionally, because microglia have been more neuroprotective in female than male mice [33], further research should determine whether variabilities in glial populations between sexes affect GBM tumorigenesis in humans.

Abnormal antitumor T-cell responses are common in GBM, which suggests that immunosuppression is being actively induced. “*Myeloid-derived suppressor cells (MDSCs) comprise a heterogeneous population of myeloid cells that are significantly expanded in cancer patients and are associated with tumor progression*” [84] and poor overall survival [85]. Myeloid-derived suppressor cells “*inhibit cytotoxic responses mediated by natural killer cells and block the activation of tumor-reactive CD4^+^ T helper cells and cytotoxic CD8^+^ T cells*” [86]. Elevated levels of MDSCs are found in the peripheral circulation of patients with GBM, are a major component of the tumor’s microenvironment and an increased tumor infiltration of MDSCs is associated with a poor outcome [87,88,89]. MDSCs are subclassified into monocytic (mMDSC) and granulocytic (gMDSC), both delay the activity of natural killer cells, mMDSCs play a role in the maintenance of primary tumors and gMDSCs promote metastasis [90,91]. A study by Bayik et al. proved that MDSCs levels differ between male and female GBM mice, males having an enriched mMDSC environment and females elevated gMDSC blood levels, both associated with a poor prognosis independently [92]. This same study demonstrated the impact that targeted therapies against these MDSCs have depending on if you are male or female. Male GBM mice can be targeted with anti-proliferative agents against mMDSCs, whereas female mice therapies can inhibit gMDSC via blocking the IL-1B pathway (canakinumab) [88,92]. Future clinical trials should use in their favor the recent advances that MDSCs play on GBM environment and how sex specific these findings are to guide future therapies. An example could be the use of canakinumab (monoclonal antibody against IL-1B) which has demonstrated a reduction in lung cancer incidence withing atherosclerotic patients [93]. Regulatory T cells in tumor-bearing brains could also be partly responsible for the ineffective immune responses to GBM [81,85]. Further studies should take into account the genetic profile of both GAMs and T cells within and around the tumor. Such studies should focus on evaluating specific sex differences that may contribute to GBM microenvironmental modulation.

Additionally, T-cell infiltration occurs within malignant astrocytomas, and the presence of tumor-infiltrating lymphocytes might predict clinical outcomes [34]. The CD4^+^ T_H_2 subtype is observed in patients with advanced disease and may contribute to poor therapy response in GBM patients [30]. Patients immunopositive for CD8 have a significantly higher survival rate than those immunonegative for CD8 [34]. These data suggest that patients with GBM with an anti-inflammatory immune profile could have a worse prognosis than those with a proinflammatory immune profile.

Of importance, although sex-specific immune differences are not well known in GBM, certain differences do exist, especially in T lymphocytes, in multiple CNS pathologic processes including stroke, CNS autoimmune disorders, and Parkinson and Alzheimer disease. Stroke is sexually dimorphic, and T lymphocytes have a critical role in stroke outcome and response. For example, women with stroke show lower regulatory T-cell efficacy to modulate immune response after stroke [94]. In contrast, mouse models of middle cerebral artery occlusions have identified that IL-4 is required for female neuroprotection during the estrus phase of the estrous cycle [95], that CD4^+^ cells are higher in males while CD8^+^ cells are higher in females [31], and that splenectomy decreases infarct volume and neuroinflammation in males but not females [96]. All these immunologic differences, together with estrogen being considered a neuroprotective hormone, make women more stroke resistant [97]. Therefore, we can make an interesting connection between the pathologic mechanisms of stroke and GBM. If high levels of CD8^+^ cells are found in female mice with stroke and immunopositive CD8^+^ patients with GBM have better survival, we can infer that women with GBM probably have higher levels of CD8^+^ cells than men.

In CNS autoimmune disorders, as with all autoimmune disorders, women are more commonly affected, but no clear explanation for this is yet known. Even though hormones could have a role, T lymphocyte and immune system differences are being studied, with interesting results and associations. In multiple sclerosis, women have increased rates of disease incidence, prevalence, and relapse. Although there is no exact explanation for these differences, variations in T-lymphocyte DNA methylation of the *Foxp3* gene on the X chromosome suggest that maternal vs. paternal imprinting underlies sex differences in autoimmunity [98]. Women also show higher inflammatory activity on radiologic studies, and immune challenges and autoantigen-specific responses are stronger in women than in men [35]. On the basis of the higher inflammatory activity in women with multiple sclerosis compared with men, and knowing that an anti-inflammatory immune response shows a worse prognosis than does a proinflammatory immune profile, we can assume that women with GBM have a higher proinflammatory profile than men and, therefore, a better outcome.

Neuromyelitis optica, an autoimmune disease in which patients have development of autoantibodies against the astrocyte water channel aquaporin-4, is also more common in women, and they are diagnosed at a younger age than men. This sex difference depends on the antibody status (the female to male ratio is 9:1 in seropositive patients); therefore, an immune system difference exists between women and men [35]. In mouse models of autoimmune encephalomyelitis, mast cells from female strains produced IL-33 in response to testosterone and led to a type 2 immune shift response that is considered protective [99].

The prevalence of Parkinson disease is greater in men, and they have an earlier age of onset. The main difference between men and women has been found in the nigrostriatal dopaminergic pathway, which arises from hormonal, immunologic, and genetic differences [100]. In terms of specific immune differences, excessive glial activation and failure to resolve neuroinflammation response can exacerbate Parkinson disease. For example, neonatal male rats have more microglia in the hippocampus and brain regions focused on cognition and memory than females do, and adult female rats have more microglia than males in the same brain regions. These differences may be associated with vulnerabilities to certain diseases, including Parkinson and Alzheimer disease [101].

Similar to GBM, Parkinson disease demonstrates the important role of microglia in the pathogenesis of the diseases and differences between men and women. Therefore, future GBM studies to understand sex differences must focus on microglial differences and their role. With regard to Alzheimer disease, mouse studies show that males no longer show plaques after 1 year, but early in life they exhibit greater signs of systemic autoimmunity compared with females. Depleted CD^+^ T splenocytes also increased autoantibody levels in males but not in females, which indicates that systemic autoimmunity is worse in males [35].

In contrast, testosterone has been hypothesized to suppress T_H_2 cell responses in men, whereas estrogens enhance T_H2_ and suppress T_H_1 responses in women. Thus, it appears that sex hormones may affect the strength of immune responses in opposite directions [102], which could have important effects in the GBM microenvironment which must be studied further. Because the microenvironment has an important role in GBM proliferation and survival [103], it could possibly be involved in the GBM-specific sex differences in outcome, clinical features, and prognosis.

Although the immune response against GBM has been well established during the past several decades, it is clear that there is still a lack of evidence regarding sex-specific differences in the GBM immunologic microenvironment. Immunologic mechanisms driving sex differences in GBM are complex and affected by hormonal, genetic, and molecular differences. Whereas estrogens and testosterone tend to have a proinflammatory effect in men and an anti-inflammatory effect in women, stroke and multiple sclerosis models show that women have a proinflammatory state and better outcomes in these diseases. More attention should be given to studying sex-specific immunologic differences in CNS pathologic processes, especially in malignant brain tumors in which little is known. Evaluation of sex differences in GAMs, T lymphocytes, cytokine secretion, and glial cells are needed to expand the field of sex-specific immune differences with the goal of understanding GBM pathogenesis and possible treatment strategies.

Last but not least, even though hormones play a role on GBM sex differences, it is important to mention the impact that age has on GBM diagnosis and sex differences. A median age of diagnosis of GBM is 64 years of age and as mentioned before it is more frequent in males than females even among elderly patients [104]. This age of diagnosis brings up an interesting point, most women at this age are in their post-menopausal stages of life, therefore hormones may not play such a profound role on GBM pathology and sex difference on elderly patients. Different factors changing while aging, specifically immunological CNS changes, explain why these sex differences exist on elderly patients.

As we age, multiple immunological changes occur, especially an enhanced global immunosuppression which may initiate GBM pathology. It is hypothesized that this global immunosuppression contributes to GBM cell initiation. A study by Ladomersky et al. found that between 60–69 years of age (most common age for GBM diagnosis) CD4^+^ T cell levels are maximal due to an increase of immunosuppressive regulatory T cells such as T regs within the same age group, additionally, CD8^+^ T cells decrease progressively while aging [105]. These changes cause a decrease on CD8^+^/Treg ratio which is associated with a diminished overall survival. Interestingly these findings correlate with the mean age of diagnosis for GBM and could explain the higher frequency in the elderly population. A similar study by the same group demonstrated that immunosuppressive IDO1 is significantly increased in the healthy brains of 73-week-old mice (considered as aging mice) as well as in normal human brain among 60–69 years of age [106,107]. Elevated levels of PD-L1 and CD11c (marker associated with immune sentinel dendritic cells) were also higher in the brain of patients between the same age range [105]. This brain immunosuppression seen on the elderly patients may explain the reason why GBM patient survival is decreased regardless of the treatment with immune-checkpoint inhibitors, which have had remarkable survival benefits in multiple cancers [108]. This immunosuppression is limited to the brain and having its highest peak between the ages of 60–69 it explains the important role that it has on GBM pathology as it correlates with the age of GBM diagnosis (64 years of age). This demonstrates that an increased brain immunosuppression may be the leading initiating factor of GBM pathology in the elderly and that future therapies must focus on this area. Additionally, at the molecular level in the mesenchymal GBM subtype there is a male to female ratio of 2:1 in the loss of function in NF1, PTEN and TP53 [29,109]. These immunological differences give an explanation on why GBM presents more often in the elderly and the worst prognosis these patients have. Adding the molecular differences between sex, it explains why male tend to have a higher frequency and worse prognosis than female even on elderly patients. Future research must be done to know the specific immunological brain differences that exist between male and female, especially levels of CD4+, CD8+, IDO1 and PD-L1 in the brains of patients with GBM.

### 3.5. Genetic and Molecular Mechanisms

Sexually dimorphic mechanisms have a major impact on cancer biology and gene expression in brain tumors, such as GBM. Evidence suggests that cell-intrinsic sex differences in gene expression may underlie the predominance of GBM in men [109] and could provide novel markers with prognostic and possibly therapeutic relevance in GBM [38].

The mechanisms by which sex might affect GBM progression range from the cellular to the organismal level [29,64]. Regardless of sex, GBM can be classified into 4 transcriptionally separated molecular subtypes: mesenchymal, neural, proneural, and classical. The mesenchymal subtype is characterized by the downregulation of neurofibromin (*NF1*), *PTEN*, and *TP53*. Interestingly, the downregulation of these genes in the mesenchymal subtype exhibits the greatest discrepancy in male to female ratio (2:1). The proneural and neural subtypes also exhibit differences by sex similar to those observed in the mesenchymal subtype. In contrast, the classical subtype, characterized by loss-of-function gene variations of *CDKN2A* [104], occurs with equal incidence in men and women [29].

Given the highly important role of p53 function for tumor suppression and maintenance of the normal cell cycle, evidence shows that sexual dimorphism could affect the p53 pathway, resulting in differences in GBM transformation by sex [29]. Male and female astrocytes with loss-of-function variations of *NF1* (*NF1*^−/−^) and p53 (*DNp53*) in the mesenchymal GBM model have significantly different tumor growth rates and clonogenic potential [109]. In the same study, male *NF1*^−/−^ *DNp53* astrocytes grew 4 times faster and had a higher clonogenic cell frequency than female *NF1*^−/−^ *DNp53* astrocytes. These sex-specific molecular differences contributed to unequal in vivo tumorigenesis. That study also showed that the tumor suppressor gene *RB1* has an important role because it exhibited greater inactivation in male GBM astrocytes than female GBM astrocytes. This sexually dimorphic activity of *RB1* in astrocytes was also shown by similar tumor growth rates among male and female astrocytes after the complete depletion of both RB1 and p53 function. Thus, when RB1 function remains intact, sex differences have distinct effects in cancer biology [109].

To further investigate the mechanisms by which female cells protect themselves upon loss of NF1 and p53 function, cyclin-dependent kinase inhibitors were studied in murine GBM astrocytes. Interestingly, cyclin-dependent kinase inhibitors such as p16, p21, and p27 were found to contribute to sex differences in RB1 regulation, tumorigenesis, and response to DNA damage [37]. Female GBM tumors expressed greater levels of p21 and p27 and a greater increase in p21 mRNA and protein levels than male GBM astrocytes in response to chemotherapy with etoposide, a topoisomerase II inhibitor. Surprisingly, the combined loss of p16, p21, and p27 made female cells as competent for in vivo tumorigenesis as male cells. These findings suggest that negative growth regulators contribute to sex differences in GBM. In addition, higher expression of the tumor suppressor genes *Btg2* and *p63* was seen in female GBM astrocytes, whereas higher expression of genes involved in tumor progression and invasion (*Hmga2* and *shh*) was seen in male GBM astrocytes [37]. These data suggest that a GBM survival advantage in women might be due to the maintenance of normal cell cycle regulators and genome stability. However, the correlation between these cell cycle regulators and sex-specific GBM overall survival still needs to be addressed.

Another signaling pathway found to be different between men and women with GBM is the cyclic AMP (cAMP) pathway. cAMP levels modify GBM risk differently by sex in patients with neurofibromatosis type 1 (NF-1) and are also determinants for gliomagenesis. Single-nucleotide polymorphisms (SNPs) in cAMP pathway components increase the risk of glioma in female NF-1 patients and suppress the risk in male patients [110]. These data indicate that sex might be involved in the contribution of the cAMP pathway to gliomagenesis in NF-1 patients, and therefore men and women might respond differently to cAMP signaling pathway–related drugs [64].

A recent sex-stratified genome-wide association study in glioma identified three glioma risk loci that differ between men and women with GBM [111]. Thus, these three SNPs could explain GBM phenotypic variance between sexes. Another recent study suggested that sex-specific differences might affect the relationship between SNPs and GBM phenotype. That study found 25 genes within five regions in men and 19 genes within six regions in women associated with GBM risk. Interestingly, the expression of epidermal growth factor receptor was significantly associated with GBM in men, whereas there was a female-specific association with telomerase reverse transcriptase expression for GBM risk. Additional analyses using gene- and pathway-based targets may further delineate sex differences regarding inherited GBM risk [112] and could provide the mechanisms underlying the sex-specific differences in gliomagenesis.

Epigenetic modifications, in particular the methylation status of the O^6^-methylguanine-DNA methyltransferase (MGMT) promoter, have significant clinical relevance in determining GBM disease outcome. Evidence shows that 80% of women with GBM have hypermethylated MGMT promoter status, vs. only 27% in men. Since MGMT promoter hypermethylation is associated with better chemotherapy response, this may explain why women generally respond better to chemotherapy plus radiotherapy and have a better outcome in GBM than men [15,40] and suggests that sex differences in gene expression are important for survival and therapy response. Moreover, a recent study analyzed therapeutic responses between male and female patients with GBM using magnetic resonance imaging and computational algorithms for calculating sex differences in GBM growth velocities and the transcriptome. In that study, standard treatment was more effective in women than in men, and better survival in men was correlated with downregulation of cell cycle regulator genes, whereas in women it was correlated with downregulation of integrin signaling pathway components [7]. Because cell cycle regulator genes are important for cell proliferation and the integrin signaling pathway components are necessary for cell migration, this could suggest a higher proliferative rate in men and a higher migration rate in women with GBM. We can speculate that by designing sex-specific targets, such as blocking cell cycle regulators in men and integrin signaling pathway components in women with GBM, we could achieve significant improvements in GBM outcome and therapy response.

The same authors reported that sex differences in tumor phenotype are dependent on differential Brd4-bound enhancer regulation of stem cell–like phenotypes in male and female, both in mice and human GBM cells; and that inhibition of Brd4 resulted in decreased in vitro clonogenicity and in vivo growth of male tumors and opposite effects on female cells and tumors [113]. Sex-specific transcriptome expression in GBM patients has been studied recently. With use of transcriptome data available through The Cancer Genome Atlas, one study identified five male and five female clusters related to GBM survival [7]. This study concluded that these clusters differ between men and women and were related to disease-free survival. A mutation in isocitrate dehydrogenase 1 (IDH1) stratified survival among male clusters with this particular variant, whereas the survival advantage of the female cluster was independent of the IDH1 variation status. These data suggest that the *IDH1* sequence variation interacts differently in men and women [7]. In addition, some studies indicate that *IDH1* sequence variation is a better prognostic marker in male than in female patients with GBM [38,39] and might exhibit a sex-specific survival benefit for men that will require further evaluation. Interestingly, there is a sex-specific cluster difference in GBM molecular subtypes and their outcome. The neural, proneural, and mesenchymal subtypes exhibit lower incidence and higher survival in women than in men, and this is related to cluster differences between sexes. In contrast, neither male nor female cluster differences are evident for the classical subtype of GBM [7], the only molecular subtype in which there is no sex difference in incidence [64]. Recent studies indicate that even genes with similar expression in men and women could have different effects in therapy response and survival [7]. These data suggest that differences in GBM survival between men and women might be associated not only with differences in gene expression but also with gene effect and that sex effects in GBM are mediated not only by sex hormones but also by tumor cell intrinsic sex differences.

Prognostic biomarkers in GBM, such as status of the Wnt receptor Frizzled-7 (FZD7), MGMT, and IDH1, have been recently studied in men and women separately. FZD7 promotes tumor progression, and high expression of FZD7 is associated with poor survival only in male GBM patients [38]. Interestingly, FZD7 currently can be targeted by monoclonal antibody therapy [114]. As already mentioned, sequence variation of *IDH1* is a better prognostic marker and is significantly associated with longer survival in men with GBM than in women [38,39], whereas the methylated MGMT promoter is more common and is associated with longer survival only in women [15,38,40]. These novel sex-specific biomarkers could have an important role as prognostic factors and therapeutic targets for GBM [115].

The potential effect of sex-specific genotype has also been studied in pediatric brain tumors. Polymorphic variants across genes have been shown to modify the risk and development of pediatric brain tumors [116,117]. Glutathione S-transferase mu 1 gene deletion and its dual deletion with the glutathione S-transferase theta 1 gene were found to be strongly associated with brain tumor in males, including tumors of glial origin. In contrast, the increased genotype-specific risk of glial tumors in females was associated with the expression of *CYP1A1* [118]. Despite divergent pathogenesis, it is interesting that male predominance in GBM exists in both adult and pediatric patients [64]. We still do not know whether SNPs differ between pediatric and adult GBM patients and how differences in sex-specific gene expression contribute to GBM growth at different stages of life.

**Figure 2:**
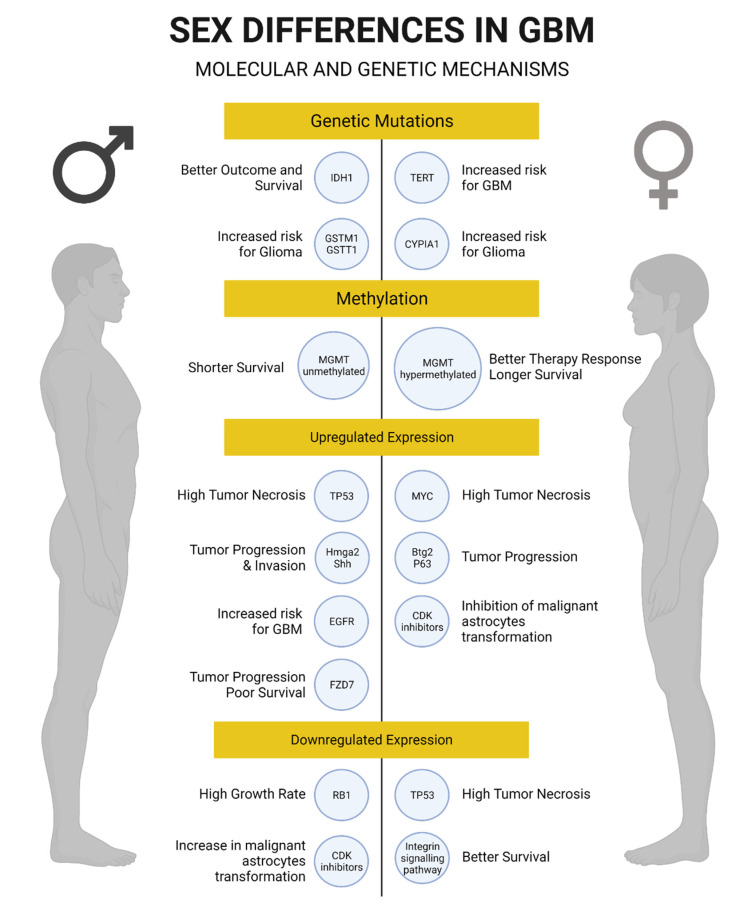
Summary of genetic and molecular mechanisms associated with glioblastoma in men and women. Created with BioRender.com.

One area of cancer research that has been studied in depth is telomere length, which is known to be affected by both age and sex. Previous studies suggested that men have shorter telomeres and higher attrition rates with aging than women do [119]. Analysis of SNPs associated with telomere length in peripheral blood leukocytes found that telomeres are approximately 5% longer in patients with glioma [120], which is associated with an increased glioma risk [121]. Thus, there may be a relationship between sex-specific variations in telomere length and increased risk of GBM. Overall, these recent findings suggest that sexual dimorphism is an important factor in GBM susceptibility (Figure 2). Therefore, further studies should evaluate how distinct aspects of gene expression and their effects between men and women affect GBM tumorigenesis. This could enhance our understanding for developing new approaches to sex-specific targets and prognostic biomarkers. Moreover, in vivo studies that yield insights into sex-specific effects in GBM therapy response are needed before designing further clinical trials.

### 3.6. Outcomes

As already mentioned, women with GBM have a better outcome than men. In fact, being female is a prognostic factor for better survival after concurrent chemoradiotherapy [122]. This sex-specific feature correlates with MGMT promoter methylation status. Hypermethylation of the MGMT promoter region has been suggested to be more common in women than men. Therefore, this epigenetic modification and its sex-specific effect could explain the favorable outcome observed in women with GBM [40,123]. However, women more often have severe hematologic toxicity when treated with radiotherapy plus temozolomide, and this has also been correlated with MGMT promoter hypermethylation [122,124]. Surprisingly, women have a significantly higher risk of development of a secondary brain cancer if treated with radiotherapy [41], although this has not yet been related to MGMT promoter methylation status, and further studies should address this relationship. Moreover, a recent study evaluating tumor growth velocities showed that women with GBM have a steady and significant decline in growth velocity during temozolomide treatment compared with that in men. These data suggest that women with GBM may benefit more from standard treatment than men and that this sex difference in therapy response may contribute to the female survival advantage [7].

Although sex hormones have been reported to confer differences in treatment response and outcome among men and women with GBM [8,14], recent studies also show that cell intrinsic sex-specific features are determinants for these same circumstances (treatment and outcome) [7]. Thus, clinical, molecular, and genetic factors should be considered individually when offering specific GBM therapy for men and women. Further clinical trials should be aware of these variations in treatment response when studying sex-specific effects in GBM.

### 3.7. Neurogenic Niches

Neural stem cells (NSCs) are multipotent stem cells that reside in the SVZ and subgranular zone of the hippocampus. The SVZ is the largest neurogenic niche of the mammalian brain, and the resident NSCs are able to self-renew as well as to generate neural progenitor cells (NPCs). NPCs can migrate across the rostral migratory stream toward the olfactory bulb, where they differentiate into mature interneurons. Malignant brain tumors such as GBM have a subpopulation of cells called *brain tumor stem cells* with similar stem cell characteristics to NPCs but with uncontrolled cell proliferation. These brain tumor stem cells are believed to lead to tumor recurrence at distal sites from the original tumor location, particularly due to their high migratory capacity [125]. In fact, contact of GBM with the SVZ correlates with lower overall survival, lower progression-free survival, and early recurrence [126,127,128,129,130]. Because men more frequently have recurrence [131] and GBM proximal to the ventricle has a greater incidence in men than women [22], further studies should evaluate sex-specific differences in neurogenic niches between men and women and how they contribute to GBM survival and recurrence.

NSCs enhance the brain’s capacity to respond to environmental changes and to replace damaged cells after injury [43]. Evidence suggests that gonadal hormone exposure, particularly estrogens, might affect neurogenesis by increasing cell proliferation in the adult neurogenic niches [132,133,134,135]. In fact, exogenous estrogen administration increases hippocampal neurogenesis in female rats after cerebral ischemia [136]. Moreover, estrogens selectively mobilize NSCs in the third ventricle, which could relate to their neuroprotective effect [43]. Testosterone also has a strong sex-specific transcriptional effect on NSCs and NPCs, altering gene expression in both sexes through transcriptional and epigenetic modifications [42]. These findings suggest that both sex hormones cause cellular changes that might contribute to sex-specific differences in the adult neurogenic niches. In contrast, sexual dimorphism in gene expression contributes to sex differences in NSC proliferation, even in the absence of gonadal hormones such as testosterone [42]. Therefore, differences in NSCs between men and women might be due to both hormonal and cell intrinsic effects.

Recent evidence has shown that cerebrospinal fluid (CSF) modulates migration of NSCs in the brain during development. One clear example is the importance of the Slit/Robo signaling pathway, which has various functions such as angiogenesis, axon guidance, and cell migration. Within this signaling pathway, Slit ligands are produced in the choroid plexus and released to the CSF to exert their chemorepulsive effects. The role of activation of this pathway has also been investigated in the context of brain tumors, based on the similarities between NSCs and brain tumor stem cells. When Slit ligands interact with their Robo receptors, tumor migration increases significantly. Another example associating CSF with tumor progression is that neural cell adhesion molecules, which are cell surface receptors broadly expressed in the CNS, are highly expressed in the CSF and correlate with meningeal spreading of medulloblastomas [125]. CSF has been previously associated with tumor migration, but studies proving a significant difference in the influence of CSF between male and female in brain tumors are limited [137]. In addition, few reports have examined differences between sexes in CSF composition in health and disease.

There is minimal evidence to compare differences in neurogenic niches between men and women, so the hypothesis that sex differences in NSCs and CSF composition contribute to differential GBM tumorigenesis and sex-specific survival warrants further investigation.

## 4. Conclusions

The ways in which men and women differ have significant importance for human health and disease. This review has focused on the recent evidence indicating that GBM is a sexually dimorphic disease. Personalized studies to achieve sex-specific targeting in GBM will require identifying and understanding the underlying genetic and molecular mechanisms by which GBM differs between sexes. Thus, preclinical studies should first be conducted in male and female separately before translating their results into GBM clinical trials.

It is of great importance that the treatment approach for patients with GBM include distinction between men and women with the purpose of creating sex-specific therapies to improve the overall outcome for each patient. Much research has focused on sex differences in GBM in terms of pathophysiology, cancer biology, hormones, metabolism, tumor location, clinical presentation, treatment response, recurrence, and outcome, as well as the role of CSF and NSCs in GBM. Nevertheless, both preclinical and clinical future studies must be encouraged in these areas to better distinguish the sex-specific differences between men and women with GBM.

## Figures and Tables

**Table 1:** Sex Differences in GBM.

	Men	Women
Clinical Features	Location: Left temporal lobe and periventricular frontal region [21]; higher incidence in frontal than temporal lobe [22] Subtype: Primary tumors [3,15]	Location:Right temporal lobe and periventricular frontal region [21]; higher incidence in temporal than frontal lobe [22] Subtype: Secondary tumors [3,15]
Hormones	Higher testosterone levels and androgen receptors associated with higher incidence [23]	Greater incidence during menopause and post menopause [8], with longer survival in premenopausal period [14] Increased risk with late menarche and early menopause [24] Protective effect of estrogens [25]
Metabolism	DM2 decreases risk [26,27] Glycolytic gene overexpression decreases survival [28] Larger body size and rapid development associated with greater cancer risk [29]	No relationship with DM2 decreasing risk Glycolytic gene overexpression increases survival [28]
Immune System	Higher levels of IL-1B, IL-6, TNF-α (no known effect yet) [29] CD4^+^ shows worse a prognosis in GBM (CD4^+^ is higher in men with stroke) [30,31] Anti-inflammatory environment shows a worse prognosis in high-grade gliomas (anti-inflammatory state seen in men with multiple sclerosis) [32]	Microglia are more neuroprotective in female mice (no known effect yet) [33] CD8^+^ shows higher survival in GBM (CD8^+^ is higher in women with stroke) [31,34] Proinflammatory environment shows a better prognosis in high-grade gliomas (proinflammatory state seen in women with multiple sclerosis) [35]
Genetic and Molecular Mechanisms	Higher incidence in mesenchymal, neural, and proneural subtype [29] *NF1* inactivation → greater growth *RB1* greater inactivation [36] Greater expression of Hmga2 and Shh [37] *IDH1* sequence variation is an important prognostic marker [38,39]	*NF1* inactivation → lesser growth Greater expression of CDK inhibitors, Btg2 and p63 [37] Higher MGMT hypermethylation [15,40] *IDH1* sequence variation is not an important prognostic marker [38,39]
Outcomes	Worse outcome and worse overall survival [29]	Greater benefit from standard treatment [7] Better outcome and overall survival independent of treatment and age [1,12,13] Higher risk of a secondary brain cancer [41]
Neurogenic Niche	Greater incidence proximal to the ventricle [22] Testosterone → transcriptional effect on NSCs [42]	Estrogens mobilize NSCs → neuroprotective effect [43]

Abbreviations: CDK, cyclin-dependent kinase; DM2, type 2 diabetes; GBM, glioblastoma multiforme; IL, interleukin; MGMT, O^6^-methylguanine-DNA methyltransferase; NSCs, neural stem cells; TNF, tumor necrosis factor.

**Table 2:** Clinical Differences Between Men and Women in Glioblastoma.

Study	Parameter Studied	Men	Women
Chakrabarti et al. [19]	Anatomic subsite affected	More common at each anatomic subsite, except for the posterior fossa	Posterior fossa (similar male:female ratio)
Bilello et al. [21]	Lobe affected	Left temporal lobe and periventricular frontal region	Right temporal lobe and periventricular frontal region
Li et al. [22]	Incidence of tumor location	Higher in frontal lobe (proximal to ventricle) than temporal lobe	Higher in temporal lobe than frontal lobe

## Data Availability

Not applicable.
